# Changes in DNA Damage Response Markers with Treatment in Advanced Ovarian Cancer

**DOI:** 10.3390/cancers12030707

**Published:** 2020-03-17

**Authors:** Paul Kubelac, Catherine Genestie, Aurelie Auguste, Soizick Mesnage, Audrey Le Formal, Patricia Pautier, Sebastien Gouy, Philippe Morice, Enrica Bentivegna, Amandine Maulard, Julien Adam, Patriciu Achimas-Cadariu, Alexandra Leary

**Affiliations:** 1 Department of Oncology, Iuliu Hatieganu University of Medicine and Pharmacy, 400012 Cluj Napoca, Romania; paulkubelac@gmail.com (P.K.); patrick.achimas@hotmail.com (P.A.-C.); 2 The Oncology Institute “Prof. Dr. Ion Chiricuta”, 400015 Cluj Napoca, Romania; 3 INSERM U981, Gustave Roussy Cancer Center, 94805 Villejuif, France; catherine.genestie@gustaveroussy.fr (C.G.); aurelieauguste@gmail.com (A.A.); SoizickM@adhb.govt.nz (S.M.); audrey.leformal@gustaveroussy.fr (A.L.F.); 4 Gustave Roussy Cancer Center, Department of Pathology, Université Paris-Saclay, 94805 Villejuif, France; julien.adam@gustaveroussy.fr; 5 Gustave Roussy Cancer Center, Department of Medical Oncology, Université Paris-Saclay, 94805 Villejuif, France; patricia.pautier@gustaveroussy.fr; 6 Gustave Roussy Cancer Center, Department of Surgery, Université Paris-Saclay, 94805 Villejuif, France; sebastien.gouy@gustaveroussy.fr (S.G.); philippe.morice@gustaveroussy.fr (P.M.); enrica.bentivegna@gustaveroussy.fr (E.B.); amandine.maulard@gustaveroussy.fr (A.M.)

**Keywords:** ovarian cancer, DNA damage repair, temporal heterogeneity, survival

## Abstract

Ovarian cancer (OC) is sensitive to upfront chemotherapy, which is likely attributable to defects in DNA damage repair (DDR). Unfortunately, patients relapse and the evolution of DDR competency are poorly described. We examined the expression of proposed effectors in homologous recombination (HR: RAD51, ATM, FANCD2), error-prone non-homologous end-joining (NHEJ: 53BP1), and base excision repair pathways (BER: PAR and PARP1) in a cohort of sequential OC samples obtained at diagnosis, after neoadjuvant chemotherapy (NACT), and/or at relapse from a total of 147 patients. Immunohistochemical (IHC) expression was quantified using the H-score (0–300), where H ≤ 10 defined negativity. Before NACT, a significant number of cases lacked the expression of some effectors: 60%, 60%, and 24% were PAR-, FANCD2-, or RAD51-negative, with a reassuringly similar proportion of negative biomarkers after NACT. In multivariate analysis, there was a poorer progression-free survival (PFS) and overall survival (OS) for cases with competent HR at diagnosis (PRE-NACT 53BP1−/RAD51+, hazard ratio (HR) 3.13, *p* = 0.009 and HR 2.78, *p* = 0.024) and after NACT (POST-NACT FANCD2+/RAD51+ HR 1.89, *p* = 0.05 and HR 2.38, *p* = 0.02; POST-NACT PARP-1+/RAD51+ HR 1.79, *p* = 0.038 and HR 2.04, *p* = 0.034), reflecting proficient DNA repair. Overall, HR-competent tumors appeared to have a dismal prognosis in comparison with tumors utilizing NHEJ, as assessed either at baseline or post-NACT. Accurate knowledge of the HR status during treatment is clinically important for the efficient timing of platinum-based and targeted therapies with poly(ADP-ribose) polymerase inhibitors (PARPi).

## 1. Introduction

Ovarian cancer (OC) is the most lethal gynecological malignancy and the fifth leading cause of cancer-related death in women, with a five-year survival in advanced cases of 29% [1]. More than 60% of patients present with advanced-stage disease, whose standard treatment consists of platinum chemotherapy and cytoreductive surgery [2]. Response rates to first-line chemotherapy exceed 70% [3]; unfortunately, most patients eventually relapse within two years from diagnosis [4].

Platinum agents cause extensive DNA damage mainly by the formation of crosslinks that elicit a variety of repair mechanisms. The initial responsiveness to platinum agents in ovarian cancer is probably linked to frequent defects in the homologous recombination (HR) pathway present in approximately 50% of cases, which make tumor cells incapable of repairing platinum-induced DNA breaks and lead to the subsequent accumulation of a fatal DNA damage burden [5,6]. While BRCA1/2 mutations have emerged as robust predictors of HR defects (HRD), efforts to identify other genomic predictors of HRD such as mutations in HR genes or HR scars have failed. The expression of DNA damage repair (DDR) proteins may provide an alternative read-out for DNA repair competency. The repair of DNA breaks involves a complex interplay between DNA repair pathways. Loss of other key effectors of effective DNA break repair beyond BRCA1/2 may be relevant to platinum responsiveness and prognosis [7].

Double-strand breaks (DSB) represent the most extensive form of DNA damage that jeopardize the viability of a cell [8]. There are two major repair mechanisms of DSBs, the HR pathway and the non-homologous end-joining (NHEJ) pathway. The first one accurately restores a damaged DNA sequence using the sister chromatid as a template, whereas the latter corrects DNA damage by directly linking the two ends together, making it error-prone through the deletion of DNA sequences surrounding the initial DSB [9]. Tumors preferentially utilizing the error-prone NHEJ repair pathway may be more sensitive to platinum damage. We have previously shown that a comprehensive evaluation of DDR proteomic biomarkers, including effectors of NHEJ, significantly improved the prognostic classification of endometrial cancer [10] and ovarian cancer [11].

ATM is a protein kinase that functions as a key signal transducer by phosphorylating downstream effectors involved in HR [12,13]. RAD51 recruitment to damaged sites is instrumental in the initiation of assembly of HR repair proteins, and the resulting nucleoprotein filament searches and invades a homologous sequence [14]. Another protein with multiple functions such as strand-annealing repair of DSB, inter-strand crosslinks repair, and limitation of NHEJ, is FANCD2, a member of the Fanconi anemia gene family [15]. The protein 53BP1 channels the repair of DSB towards NHEJ by antagonizing the resection of DSBs in G1 [16]. An evaluation of the balance between HR and NHEJ may be relevant to both platinum and poly (ADP-ribose) polymerase inhibitors (PARPi) responsiveness.

PARP-1 is a highly conserved enzyme involved in numerous functions including the maintenance of genomic integrity. PARP-1 synthesizes PAR chains, negatively charged polymers used as a scaffold for the recruitment of various proteins involved in the DNA repair process, such as base excision repair (BER) [17,18].

As such, both PAR and PARP-1 expression provide a read-out for BER activity. In addition, PARPi are becoming standard of care as maintenance therapy for both first line BRCA1/2-mutated OC and platinum-sensitive relapsed high-grade OC regardless of the BRCA status [19,20,21,22]. Therefore, greater knowledge regarding the expression of the target, PARP-1, in OC throughout the disease course may become critical.

Given the complex interplay between repair pathways, a comprehensive assessment of DNA repair effectors may provide valuable insight into DNA repair competency and prognosis with first-line platinum treatment. Our first aim was to evaluate the relative expression of proposed key effectors of competent DNA repair (HR: RAD51, FANCD2, ATM and BER: PAR, PARP-1) as well as error-prone repair (NHEJ: 53BP1) and correlate DDR biomarkers to outcome (Table 1).

It is also equally important to acknowledge that ovarian cancer is a heterogeneous disease [25] and that its evolutionary trajectory is constantly under the selection pressure of chemotherapy [26,27]. Reversion mutations restoring BRCA wild-type function have been described after platinum and PARPi exposure. The evolution of DNA repair proteomic biomarkers under treatment-related selection pressure is poorly described, hence our second aim was to describe changes in DDR markers in a large cohort of advanced OC samples obtained at diagnosis, after NACT, and/or at relapse and evaluate their impact on outcome. One hypothesis was that platinum treatment could result in re-expression of mediators of efficient DNA repair which could account for poor outcomes in OC. As an exploratory endpoint, we evaluated stromal tumor infiltrating lymphocytes (sTILs) presence before and after chemotherapy with regard to DNA repair competency that might influence neoantigen load and other features of the host immune response [28].

## 2. Results

A total number of 147 patients were included in the present study. Relevant clinical characteristics are presented in Table 2. All patients had advanced intraperitoneal disease, and 14.3% had stage IV disease at diagnosis. Patients received a median of four cycles of platinum-based neoadjuvant chemotherapy (NACT), aiming for complete cytoreduction at interval debulking surgery (IDS); however. 29.9% of patients were still unresectable following the evaluation laparoscopy and were treated with chemotherapy alone. The mean gap between the last cycle of chemotherapy and surgery was 28.14 days (standard error of mean 1.34 days).

Among the clinical variables that influenced survival, in univariate analysis, only completeness of surgery had a significant impact on both progression-free survival (PFS, HR 2.94, 95%CI 1.96–4.35, *p* < 0.001) and overall survival (OS, HR 2.22, 95%CI 1.43–3.45, *p* < 0.001) for resection with no residual macroscopic disease vs. incomplete surgery or no surgery.

Immunohistochemical (IHC) markers are sensitive to sample handling and processing. The correlation between individual DDR markers was reassuringly low (e.g., Pearson r coefficient for PRE-NACT H-score PAR with PRE H-score PARP-1, PRE H-score ATM, PRE H-score 53BP1, PRE H-score RAD51, PRE H-score FANCD2 was 0.15, 0.18, 0.09, 0.17, and 0.18, respectively), supporting that variations in expression levels were unlikely to be attributable to poor pre-analytical conditions.

Before NACT, a significant number of cases lacked the expression of DDR markers: 60%, 60%, 24%, 21%, and 14.8% were PAR-, FANCD2-, RAD51-, ATM-, or 53BP1-negative (H-score ≤ 10), respectively; however only 3% lacked PARP1 expression (Table 3, Table S1).

Following neoadjuvant platinum-based chemotherapy, there was an increase in the proportion of marker-negative tumors, which was statistically significant for PAR and RAD51 (*p* < 0.01). At relapse, there was a trend for the rates of marker-negative tumors to return to baseline levels, with the notable exception of 53BP1, for which the proportion of 53BP1-negative tumors increased from 15% at baseline to 27% at relapse, with possible relevance to PARPi responsiveness at relapse.

Investigating the prognostic value of single DDR markers, in univariate and multivariate analysis (Tables S2 and S3), PRE-NACT 53BP1 and POST-NACT ATM-positive expression was associated with an improved PFS compared to cases with absent 53BP1 or ATM expression (multivariate HR 0.24, *p* < 0.001 and HR 0.54, *p* = 0.012, respectively). In multivariate analysis, PRE-NACT PAR-positive expression was associated with a worse PFS compared with absent PAR expression (HR 1.67, *p* = 0.037). No correlation with OS was found.

The biological relevance of DNA repair biomarkers is probably binary, i.e., it regards their expression or lack thereof; therefore, we were particularly interested in investigating whether conversion from positive to negative expression after chemotherapy was prognostic in paired samples. In univariate and multivariate analysis, loss of ATM after NACT was associated with significantly worse PFS and OS (multivariate HR 0.21, *p* = 0.003 and HR 0.21, *p* = 0.008), while loss of RAD51 after NACT was associated with improved PFS and OS (multivariate HR 2.55, *p* = 0.029 and HR 5.44, *p* = 0.008), as presented in Table S2 and Table 4.

**Figure 1:**
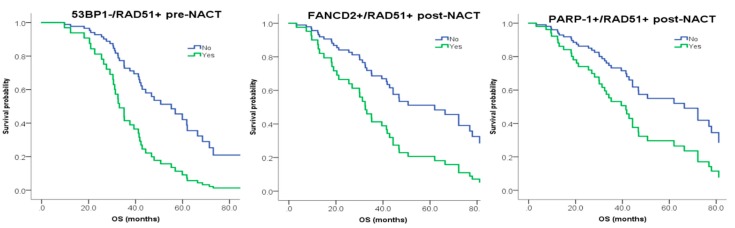
Overall survival of combined DNA repair biomarkers at diagnosis pre-NACT or post-NACT in multivariate analysis.

Given the redundancy of DNA repair pathways, a single marker might not be fully informative of the overall DNA repair competency; therefore, we next evaluated the prognostic value of combined biomarkers, before and after NACT. Cases with competent HR (RAD51+) but defective error-prone NHEJ (53BP1−) at diagnosis demonstrated significantly worst PFS and OS on multivariate analysis (53BP1−/RAD51+, HR 3.13, *p* = 0.009 and HR 2.78, *p* = 0.024). Similarly, patients with dual expression of HR biomarkers post-NACT (FANCD2+/RAD51+, HR 1.89, *p* = 0.05 and HR 2.38, *p* = 0.02) or dual expression of BER and HR biomarkers (PARP-1+/RAD51+, HR 1.79, *p* = 0.038 and HR 2.04, *p* = 0.034) had a worse outcome in terms of PFS and OS even after adjusting for completeness of surgery (Figure 1).

When analyzing sTILs expression pattern, we found an increase after NACT for all DDR subgroups (range 5.5–16.4 absolute % difference). Tumors expressing error-prone NHEJ repair biomarkers (53BP1+) had a higher mean sTILs % compared with 53BP1-negative counterparts, both pre- (26.9% vs. 15.9% sTILs) and post- (32.9% vs. 28.9% sTILs) NACT, although not reaching statistically significant levels. On the contrary, tumors expressing proficient HR (RAD51+) had a lower mean sTILs % compared with RAD51-negative cases, both pre- (19.4% vs. 24.7% sTILs) and post- (27.6% vs. 41.1% sTILs, *p* = 0.025) NACT. According to PAR and ATM biomarker status there were only minor sTILs variations (<5%), while for PARP no conclusions could be drawn due to the small number of patients. Interestingly, FANCD2-positive samples had a higher mean sTILs % compared with FANCD2-negative cases, both pre- (26.4% vs. 19.5% sTILs) and post- (33.7% vs. 26.4% sTILs) NACT, as shown in Table S4.

## 3. Discussion

Platinum agents remain the cornerstone of OC management. and the availability of PARPi has now added a significant therapeutic strategy in the armamentarium. However, beyond histology and BRCA mutations, robust biomarkers of platinum and PARPi sensitivity still elude us. A number of studies have evaluated the prognostic and predictive value of genomic markers of HRD. To date, neither mutations in HR genes (beyond BRCA1/2) nor various assays for HRD scars have been reproducibly associated with improved outcomes. In addition, as PARPi are currently mainly proposed as maintenance treatment after platinum, a real-time read-out of DNA repair competency post-platinum might be informative [7].

In the ARIEL2 Part 1 study, an exploratory analysis showed that loss of heterozygosity (LOH) classification based on a next-generation sequencing assay was highly concordant (R = 0.86, *p* < 0.0001) between paired samples obtained at diagnosis and before trial enrollment at relapse, indicative of a stable genomic signature [29]. Similar results were reported with a 3-biomarker HRD score that was based on the integration of three independent measures—LOH, telomeric–allelic imbalance, and large-scale state transitions. The authors concluded that the three-biomarker HRD score was stable in paired primary and recurrent tumor samples, suggesting that testing recurrent high-grade serous ovarian cancer tumors would not alter their management [30]. However, the two aforementioned biomarkers quantify the degree of genomic scarring, which is likely static and will remain identical even if there is a change in HR competency, hence their accuracy might be limited to initial diagnosis.

In contrast, a study that examined HR gene expression of two cohorts that underwent primary debulking surgery or NACT followed by IDS revealed two distinct HR profiles that were associated with outcome after exposure to platinum-based therapy [31]. Similarly, there was an increase in BRCA1/2 protein expression at disease recurrence in paired ovarian carcinoma specimens, suggesting that an evaluation of protein expression may represent a more accurate and contemporary measure of HR competency than any genomic scar [32].

We therefore sought to describe the proteomic expression of DNA repair effectors at diagnosis and their change with first-line platinum neoadjuvant chemotherapy and/or relapse in a large cohort of OC patients. Overall, at diagnosis a significant proportion of OC tumors lacked the expression of effectors implicated in efficient DNA repair (FANCD2: 60%; PAR: 60%; RAD51: 24%; ATM: 21%), which could account for initial platinum sensitivity. At diagnosis, the loss of individual efficient (HR and BER) DNA repair biomarkers did not correlate with OS on multivariate analysis. Two DDR markers correlated with PFS alone. Loss of PAR, an effector of BER-mediated repair, showed a correlation with improved PFS but not OS on multivariate analysis. The protein 53BP1 is an important regulator of DSB repair by promoting error-prone NHEJ while inhibiting HR. The presence of 53BP1 at diagnosis was associated with improved PFS (but not OS), indicating that an active error-prone NHEJ DNA repair pathway might make tumor cells more susceptible to platinum agents [16,33]. Of particular interest, the loss of the key downstream effector of HR, RAD51, was not associated with improved prognosis. This may be explained by the fact that the loss of RAD51 may only be biologically relevant in the setting of demonstrated DNA damage (gH2AX) in the S–G2 phase (geminin), as recently published for breast cancer [34]. In the setting of fixed tissue without information on whether tumor cells are actually trying to repair DSBs, the lack of RAD51 protein expression will not be sensitive for the diagnosis of HRD. However, the positive presence of RAD51 proteins might be informative, as a sign that the tumor is HR-proficient. Among the small subset of patients whose tumors converted from RAD51-positive to RAD51-negative post-NACT, PFS and OS were significantly improved on multivariate analysis. Tumors with a combined profile of HR competency and lack of the key effector of error-prone NHEJ (RAD51+/53BP1−) at diagnosis demonstrated significantly worst PFS and OS (*p* = 0.009; *p* = 0.024) on multivariate analysis, suggesting that efficient DSB repair capacity may protect tumor cells from DNA damage induced by platinum as well as by subsequent lines of chemotherapy.

With regard to changes associated with NACT, a reassuringly similar proportion of tumors remained negative for biomarkers of effective DNA repair. In fact, the proportion of RAD51- or PAR-negative tumors increased post-NACT, suggesting that neoadjuvant platinum chemotherapy did not enrich significantly for HR or BER competency, respectively. PARP-1, the actionable target of PARPi, was widely expressed at diagnosis in 96.6% of samples and does not significantly change after NACT. Again, an evaluation of combined DNA repair biomarkers post-NACT offered the greatest prognostic information. First, we showed that the combined positivity of FANCD2 and RAD51 (FANCD2+/RAD51+), suggesting competent HR, was associated with a worse PFS and OS [35,36]. Secondly, combining RAD51 positivity with the presence of PARP-1 positivity as a surrogate for active single-strand break (SSB) repair machinery via BER (PARP-1+/RAD51+), predicted significantly worst PFS and OS. Both scenarios underline the importance of DDR competency evaluation after an initial exposure to neoadjuvant platinum. Post-NACT evaluation of DDR biomarkers may be more informative than pre-NACT evaluation, given that pre-NACT tissue samples reflect the innate biology of cancer cells, while in post-NACT samples, chemotherapy may eliminate the chemotherapy-sensitive clones and enrich for those that drive a patient’s prognosis. Similarly, in our previous paper [37], only post-NACT sTILs (not pre-NACT sTILs) predicted platinum responsiveness (platinum-free interval >6 months), suggesting that adaptive immunogenic mechanisms during chemotherapy may have an important role in determining the outcome.

When analyzing the overall sTILs expression pattern, we found an increase after NACT, as we have previously reported [37], which was consistent across all DDR subgroups. Similar reports have been published for lung cancer [38]; however, the changes induced might be dependent on the immune cell subpopulation profiles [39], possibly through immunogenic cell death and modulation of the tumor microenvironment [40]. Tumors showing HR deficiency (RAD51-negative) had a statistically significantly higher mean sTILs% compared with RAD51-positive cases, in the post-NACT setting (41% vs. 28% sTILs, *p* = 0.025), which could indicate an enhanced immune response mechanism to a higher burden of neoantigens. However, in depth additional studies are needed to characterize the dynamics of immune cells subpopulations in relation to competency of DDR pathways [41].

Looking at DDR markers expression at relapse, the number of samples was small; however, there was an increase in RAD51 and FANCD2 and a decrease in 53BP1 expression compared to post-NACT samples, which could suggest indirect evidence towards an outgrowth of resistant subpopulations at relapse with maintained HR competency and decreased expression of the more error-prone NHEJ machinery.

The limitations of this study reside in its retrospective nature, and although this is one of the first reports describing the evolution of several DDR biomarkers under chemotherapy, we could not draw any firm conclusions on the causal interactions between different DDR pathways and specific immune cell subpopulations. We have highlighted the dynamics of DDR biomarkers and their possible role in influencing outcome. However, a wider spectrum of biomarkers should be incorporated in future studies for a more accurate characterization, using a larger number of samples from spatially separate biopsies, given the heterogeneity found in ovarian cancer. 

## 4. Materials and Methods

**Figure 2:**
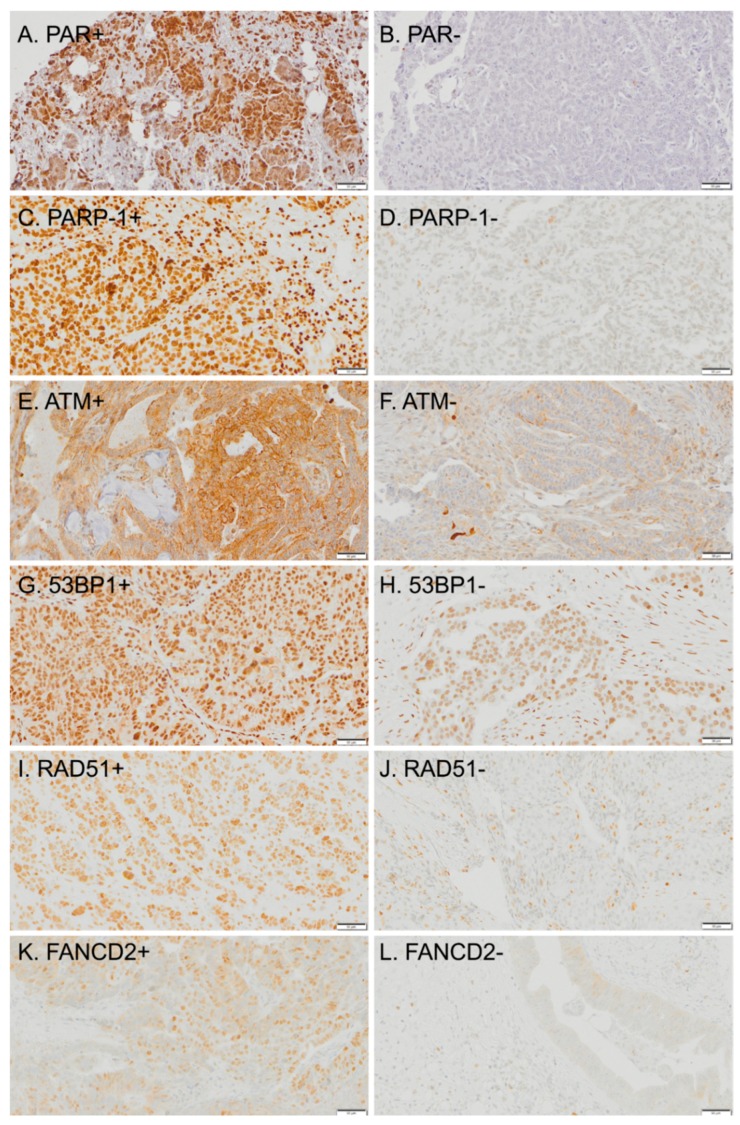
Immunohistochemical (IHC) staining pattern of DDR markers. Scale bar = 50 µm.

A cohort of 147 patients with histologically confirmed epithelial ovarian cancer treated at the Gustave Roussy Cancer Center between 2002 and 2014 were included in the study. All subjects provided signed informed consent, allowing the conduct of research on tumor samples linked to anonymized clinical data. We selected patients who underwent NACT and for whom sequential tumor tissue was available pre-, post-chemotherapy, and/or at relapse. IHC staining for all DDR markers was performed on tissue microarrays (TMAs) using three 1 mm cores selected by an experienced pathologist, with a minimum of 70% of tumor cells. The number of samples evaluable for each marker varied due to crush artifacts or because samples were lost as the TMA was increasingly sectioned. Positive controls were evaluated on lymphocytes from the same samples. IHC staining for PAR, PARP-1, ATM, 53BP1, RAD51, FANCD2 was performed as described in Table S5. DDR markers expression was blindly evaluated by two experienced pathologists using an H-score that was defined as the total sum of each product’s staining intensity (weak = 1, moderate = 2, strong = 3) and the corresponding percentage of positive cells (1–100), with values ranging between 0 and 300; a mean of 3 cores was used. Positive expression of a DDR marker was defined as an H-score >10. This threshold of 10 out of 300 was based on the hypothesis that only the loss of a DDR marker would have a biological relevance [42,43]. The IHC staining pattern of all DDR markers is presented in Figure 2. Correlation between ovarian cancer TMA cores and matched full slides was previously investigated in our department, providing similar results.

Stromal TILs evaluation was performed on hematoxylin–eosin-stained sections of either formalin-fixed or acetic acid/formalin/alcohol-fixed samples and was defined as the percentage of total intratumoral stromal area occupied by mononuclear cells, as previously described [44,45].

The statistical software used was IBM SPSS Statistics v22.0 and Prism v7.00. Abnormality of distribution for DDR markers was demonstrated by the Z-score, Shapiro-Wilk’s test, and visual inspection of their histograms and Q-Q plots. A Mann–Whitney U test was used to determine if there were differences in H-score expression between time points. A chi-square test for association was conducted between selected clinical variables and DDR markers positivity. Progression-free survival was calculated from the date of histological diagnosis to the first radiological or clinical progression. Median follow-up was quantified using the reversed Kaplan–Meyer method [46]. For univariate and multivariate analysis, a Cox model was used. Results were considered significant for two-tailed exact *p*-value < 0.05. The REMARK guidelines were used for an accurate description of prognostic markers [47].

## 5. Conclusions

This is one of the first studies that comprehensively evaluated DDR proteomic biomarkers in OC before and after platinum chemotherapy. Firstly, we demonstrated that OC frequently present loss of key of DNA repair effectors at diagnosis, with the notable exception of PARP-1, the direct target for PARPi. Also, reassuringly, NACT did not increase the expression of markers of efficient SSB and DSB repair, providing indirect evidence that first-line platinum treatment did not significantly select for DNA repair-competent clones.

At diagnosis, PAR loss and 53BP1 expression were predictive of improved PFS, suggesting that BER deficiency or use of the error-prone NHEJ pathway might undermine the effective repair of platinum-induced DNA damage. Importantly, a combined evaluation of biomarkers suggesting efficient DNA repair after NACT was most informative. We demonstrated that combined RAD51+/FANCD2+ or RAD51+/PARP1+ expression was a significant predictor of both poor PFS and OS on multivariate analysis. Further studies should investigate whether a comprehensive evaluation of both DNA repair competency and expression of candidate markers of PARPi resistance, such as PARP1 or 53BP1 loss, may predict differential PARPi responsiveness. As these DDR markers may be dynamic, the evaluations should be performed after NACT in the first line setting and on biopsies of relapsed disease in subsequent lines.

## Figures and Tables

**Table 1:** DNA damage repair (DDR) biomarkers and their relevance.

DDR Biomarker	Relevance	Reference
PARP-1	Polymerase inhibitors (PARP-1) binds to damaged DNA and synthetizes PAR chains, essential for base excision repair (BER) pathway	[17,18]
PAR	PARP-1 effector, PAR chains are a scaffold for the recruitment of DNA repair proteins, a measure of BER activity	[17,18]
ATM	DNA damage sensor and mediator of homologous recombination (HR). Also involved in DNA replication, cell cycle checkpoint arrest, and apoptosis, as means of coordinating several pathways during genomic stress	[12,13]
RAD51	Initiates repair protein assembly in the HR pathway	[23]
FANCD2	Member of the Fanconi anemia genes, involved in strand-annealing repair of DSB and in repairing inter-strand crosslinks Seems to limit the use of error-prone non-homologous end-joining (NHEJ), proposed inhibitory relationship with 53BP1.	[15,24]
53BP1	Promotes NHEJ and inhibits HR, favoring error-prone DNA repair	[16]

**Table 2:** Patients’ characteristics.

Variable	Number	%
**Median age (range)**	60.9 (20.8–78.8) years	
**The International Federation of Gynecology and Obstetrics (FIGO) stage (2014)**		
IIIC	126	85.7
IV	21	14.3
**BRCA status**		
Wild-type	66	44.9
BRCA 1/2 mutation	22	15.0
Unknown	59	40.1
**Outcome of IDS**		
Macroscopic complete resection	103	70.1
Not operated ^1^	44	29.9
**Histological type**		
High-grade serous	108	73.5
Low-grade serous	11	7.5
Clear cell	2	1.4
Mucinous	2	1.4
Other high-grade ^2^	24	16.3
**Relapse**	132	89.8
**Death**	92	62.6
**Median follow-up (95% CI)**	71.6 (63.8–79.3) months	
Median PFS (95% CI)	21.7 (18.4–24.9) months	
Median OS (95% CI)	44.9 (37.3–52.4) months	

^1^ deemed unresectable at evaluation laparoscopy, ^2^ other high-grade include poorly differentiated, mixed histology. PFS: progression-free survival, OS: overall survival.

**Table 3:** DDR markers expression before and after neoadjuvant chemotherapy (NACT) and at relapse.

Temporal Setting	PAR % Negative (no. neg/total)	PARP-1 % Negative (no. neg/total)	ATM % Negative (no. neg/total)	53BP1 % Negative (no. neg/total)	RAD51 % Negative (no. neg/total)	FANCD2 % Negative (no. neg/total)
Pre-NACT	60 (40/80)	3 (3/87)	21 (16/78)	15 (13/88)	24 (16/68)	60 (53/89)
Post-NACT	82 (72/88) ^1^	6 (5/85)	34 (28/82)	18 (15/83)	52 (37/71) ^1^	62 (53/86)
Relapse	60 (9/15)	0 (0/9)	21 (3/14)	27 (3/11)	20 (2/10)	50 (6/12)

^1^ Significantly higher number of negative samples compared to the pre-NACT number (*p* < 0.01, Chi-square test).

**Table 4:** Multivariate analysis of combined biomarkers.

Biomarker	Result	N	PFS		OS	
			HR (95% CI)	P	HR (95% CI)	P
POST-NACT ATM (PRE-NACT ATM+)	Negative	9	1	0.003	1	0.008
	Positive	22	0.21 (0.08–0.59)		0.21 (0.07–0.66)	
POST-NACT RAD51 (PRE-NACT RAD51+)	Negative	12	1	0.029	1	0.008
	Positive	15	2.55 (1.09–5.92)		5.44 (1.56–18.92)	
PRE-NACT 53BP1−/RAD51+	False	57	1	0.009	1	0.024
	True	7	3.13 (1.33–7.69)		2.78 (1.15–7.14)	
POST-NACT FANCD2+/RAD51+	False	50	1	0.05	1	0.02
	True	14	1.89 (1–3.57)		2.38 (1.15–5)	
POST-NACT PARP-1+/RAD51+	False	36	1	0.038	1	0.034
	True	29	1.79 (1.04–3.03)		2.04 (1.06–4)	

Multivariate analysis was adjusted for FIGO stage (2014), outcome of interval debulking surgery (IDS), and aggressive histology.

## Supplementary Materials

The following are available online at https://www.mdpi.com/2072-6694/12/3/707/s1, Table S1: DDR markers detailed expression before, after NACT, and at relapse, Table S2: Univariate analysis, Table S3: Multivariate analysis of individual biomarkers, Table S4: Stromal tumor-infiltrating lymphocyte expression in relation to chemotherapy exposure and DDR marker positivity, Table S5: List of antibodies and protocol used for immunostaining.
