# Supplementary material for: Changes in DNA Damage Response Markers with Treatment in Advanced Ovarian Cancer
Source: Cancers (Basel). 2020 Mar 17;12(3):707. doi: 10.3390/cancers12030707 (PMC7140046; doi:10.3390/cancers12030707)
Supplement: Supplementary file 1 [file cancers-12-00707-s001.pdf]

cancers

Supplementary Materials:

Changes in DNA Damage Response Markers with Treatment in Advanced Ovarian Cancer

Table S1. DDR markers detailed expression before, after NACT and at relapse.

	PAR	PARP-1	ATM	53BP1	RAD51	FANCD2
Pre NACT						
No. samples	80	87	78	88	68	89
No. positive samples (%) [1]	32 (40)	84 (96.6)	62 (79.5)	75 (85.2)	52 (76.5)	36 (40.4)
H-score Q25	0	200	30	100	20	0
H-score Q50	0	205	100	200	80	5
H-score Q75	57.5	270	225	220	142.5	45
Post NACT						
No. samples	88	85	82	83	71	86
No. positive samples (%) [1]	16 (18.2) [2]	80 (94.1)	54 (65.9)	68 (81.9)	34 (47.9) [2]	33 (38.4)
H-score Q25	0	162.5	0	80	0	0
H-score Q50	0	200	100	200	10	5
H-score Q75	0	210	240	200	90	40
Relapse						
No. samples	15	9	14	11	10	12
No. positive samples (%) [1]	6 (40)	9 (100)	11 (78.6)	8 (72.7)	8 (80)	6 (50)
H-score Q25	0	205	75	0	15	0
H-score Q50	0	240	220	200	55	10
H-score Q75	160	265	300	200	172.5	100

[1] Defined as H-score > 10, [2] significantly smaller number of positive samples compared to pre-NACT value ($p < 0.01$, Chi-square test), Q – quartile.

Table S2. Univariate analysis.

			PFS			OS		
		N	Median (mos.)	HR (95%CI)	P	Median (mos.)	HR (95%CI)	P
Age	≤ Median	75	19.3	1	0.85	44.9	1	0.76
	> Median	72	19.6	1.04 (0.73–1.45)		47.9	1.08 (0.7–1.62)	
FIGO stage (2014)	IIIC	126	18.9	1	0.55	44.9	1	0.90
	IV	21	23.2	0.87 (0.53–1.41)		44.3	0.97 (0.53–1.75)	
Outcome of IDS	Complete resection	103	22.9	1	<0.001	59.9	1	<0.001
	Not operated	44	15.2	2.94 (1.96–4.35)		34.2	2.22 (1.43–3.45)	
Aggressive histology[1]	No	19	20.9	1	0.11	68.5	1	0.11
	Yes	128	18.9	1.56 (0.9–2.78)		44.3	1.69 (0.88–3.33)	
BRCA status	BRCAwt	66	21.3	1	0.38	50.7	1	0.07
	mBRCA1/2	22	25	0.79 (0.47–1.33)		76.9	0.53 (0.26–1.06)	
PRE NACT PAR	Negative	48	21.1	1	0.12	56.9	1	0.22
	Positive	32	18	1.47 (0.91–2.33)		42.3	1.43 (0.81–2.56)	
PRE NACT PARP-1	Negative	3	15.2	1	0.23	34.2	1	0.068
	Positive	84	19.3	0.49 (0.15–1.59)		46.8	0.33 (0.1–1.09)	
PRE NACT ATM	Negative	16	18.3	1	0.65	50.7	1	0.6
	Positive	62	20.8	1.15 (0.63–2.08)		56.9	0.83 (0.41–1.69)	
PRE NACT 53BP1	Negative	13	14	1	0.003	46.8	1	0.77
	Positive	75	20.4	0.39 (0.21–0.73)		50.7	0.9 (0.42–1.92)	
PRE NACT RAD51	Negative	16	16	1	0.74	68.5	1	0.24
	Positive	52	19	1.11 (0.61–2.04)		44.3	1.56 (0.74–3.33)	
PRE NACT FANCD2	Negative	53	19	1	0.79	47.9	1	0.61
	Positive	36	18.5	0.94 (0.6–1.49)		44.9	1.16 (0.66–2.04)	
POST NACT PAR	Negative	72	20.3	1	0.5	44.3	1	0.83
	Positive	16	14	1.22 (0.69–2.17)		46.9	1.08 (0.56–2.08)	
POST NACT PARP-1	Negative	5	20.9	1	0.75	-	1	0.27
	Positive	80	18.9	1.16 (0.46–2.94)		44.3	2.22 (0.53–9.09)	
POST NACT ATM	Negative	28	15.1	1	0.016	41.5	1	0.12
	Positive	54	21.3	0.56 (0.34–0.9)		50.7	0.64 (0.36–1.15)	
POST NACT 53BP1	Negative	15	18	1	0.64	72.2	1	0.35
	Positive	68	20.9	0.88 (0.49–1.56)		46.8	1.45 (0.67–3.13)	
POST NACT RAD51	Negative	37	22.8	1	0.12	66.4	1	0.06
	Positive	34	14.8	1.47 (0.91–2.38)		39.5	1.79 (0.97–3.33)	
POST NACT FANCD2	Negative	53	20.3	1	0.97	44.3	1	0.93
	Positive	33	18.9	1 (0.63–1.56)		46.8	1 (0.56–1.72)	
POST NACT ATM (PRE NACT ATM+)	Negative	9	17.4	1	0.011	35.2	1	0.029
	Positive	22	29	0.3 (0.12–0.76)		81.1	0.32 (0.11–0.89)	
POST NACT RAD51 (PRE NACT RAD51+)	Negative	12	22.1	1	0.028	66.4	1	0.023
	Positive	15	14	2.5 (1.1–5.67)		39.3	3.55 (1.19–10.56)	
PRE NACT 53BP1−/RAD51+	False	57	19.8	1	0.035	50.7	1	0.041
	True	7	13.7	2.44 (1.08–5.56)		42.3	2.44 (1.04–5.56)	
POST NACT FANCD2+/RAD51+	False	50	20.9	1	0.067	62	1	0.05
	True	14	13.1	1.79 (0.96–3.33)		31.3	2.04 (1–4.17)	
POST NACT PARP-1+/RAD51+	False	36	22.8	1	0.012	72.2	1	0.012
	True	29	14.5	1.96 (1.16–3.33)		39.5	2.27 (1.2–4.35)	

[1] Aggressive histology includes high grade serous, mixed and poorly differentiated NOS. BRCAwt – BRCA wildtype, mBRCA1/2 – mutant BRCA 1/2 germline or somatic.

Table S3. Multivariate analysis of individual biomarkers.

			PFS		OS	
		N	HR (95% CI)	P	HR (95% CI)	P
PRE NACT PAR	Negative	48	1	0.037	1	0.23
	Positive	32	1.67 (1.03–2.7)		1.43 (0.8–2.56)	
PRE NACT PARP-1	Negative	3	1	0.81	1	0.4
	Positive	84	1.16 (0.34–4)		0.58 (0.16–2.13)	
PRE NACT ATM	Negative	16	1	0.82	1	0.51
	Positive	62	1.08 (0.58–2)		0.79 (0.38–1.64)	
PRE NACT 53BP1	Negative	13	1	<0.001	1	0.33
	Positive	75	0.24 (0.12–0.47)		0.68 (0.3–1.52)	
PRE NACT RAD51	Negative	16	1	0.93	1	0.7
	Positive	52	0.98 (0.53–1.82)		1.16 (0.53–2.56)	
PRE NACT FANCD2	Negative	53	1	0.76	1	0.67
	Positive	36	1.08 (0.67–1.75)		1.14 (0.63–2.04)	
POST NACT PAR	Negative	72	1	0.86	1	0.44
	Positive	16	0.95 (0.53–1.72)		0.76 (0.37–1.56)	
POST NACT PARP-1	Negative	5	1	0.98	1	0.43
	Positive	80	0.99 (0.39–2.5)		1.79 (0.42–7.69)	
POST NACT ATM	Negative	28	1	0.012	1	0.064
	Positive	54	0.54 (0.33–0.88)		0.57 (0.31–1.04)	
POST NACT 53BP1	Negative	15	1	0.56	1	0.42
	Positive	68	0.84 (0.46–1.54)		1.39 (0.63–3.03)	
POST NACT RAD51	Negative	37	1	0.27	1	0.12
	Positive	34	1.32 (0.81–2.17)		1.64 (0.88–3.03)	
POST NACT FANCD2	Negative	53	1	0.92	1	0.8
	Positive	33	1.03 (0.64–1.67)		1.09 (0.6–1.96)	

Multivariate analysis was adjusted for FIGO stage (2014), outcome of IDS and aggressive histology.

Table S4. Stromal tumor infiltrating lymphocytes expression in relation with chemotherapy exposure and DDR marker positivity.

DDR marker	I-score	PRE NACT			POST NACT			Absolute difference POST-PRE NACT mean sTILs %
		N	Mean sTILs %	St. Dev.	N	Mean sTILs %	St. Dev.	
PAR	0	43.0	22.3	23.1	67.0	31.4	23.0	9.1
	1	30.0	20.6	17.4	13.0	31.3	30.9	10.7
PARP	0	3.0	10.0	5.0	5.0	21.0	28.2	11.0
	1	77.0	23.1	21.9	71.0	30.9	23.5	7.8
ATM	0	13.0	23.5	25.6	26.0	28.9	22.7	5.5
	1	57.0	24.7	20.7	48.0	33.1	24.3	8.5
53BP1	0	11.0	15.9	15.5	13.0	28.9	26.8	13.0
	1	72.0	26.9	23.0	62.0	32.9	24.1	5.9
RAD51	0	15.0	24.7	24.5	33.0	41.1 [1]	24.0	16.4
	1	48.0	19.4	19.1	33.0	27.6 [1]	23.6	8.2
FANCD2	0	46.0	19.5	21.2	47.0	26.4	24.4	7.0
	1	33.0	26.4	24.1	31.0	33.7	22.1	7.3

[1] statistically significant difference (p = 0.025) between POST NACT paired I-score positive and negative samples, all other comparisons did not reach statistical significance (p > 0.05). PRE NACT mean sTILs % were calculated for PRE I-score values while POST NACT mean sTILs % were calculated for POST I-score values.

Table S5. List of antibodies and protocol used for immunostaining.

Marker	Dilution	Supplier	Machine	Species	Reference	Unmasking procedure	Incubation	Reagents
PAR	1:6000	Calbiochem	Benchmark Ultra Ventana	Mouse	AM80	CC1 95 °C	36 min	Kit Ultra-View-HRP (760-500) Hematoxiline (790-2208) Bluing Reagent (760-2037)
PARP-1	1:1000	Abd Serotec	Benchmark Ultra Ventana	Mouse	MCA1522G	CC2	44 min	Kit Ultra-View-HRP (760-500) Hematoxyline (790-2208) Bluing Reagent (760-2037)
ATM	1:150	Abcam	Manual	Rabbit	AB32420	Antigen retrieval citrate pH6.0	ON RT °C	DAB as chromogen
53BP1	1:200	Abcam	Benchmark Ultra Ventana	Rabbit	AB21083	CC1 95 °C	1 h	Kit Ultra-View-HRP (760-500) Hematoxiline (790-2208) Bluing Reagent (760-2037)
RAD51	1:100	SPRING	Benchmark Ultra Ventana	Rabbit	E19102	CC1 95 °C	92 min	Kit Ultra-View-HRP (760-500) Hematoxyline (790-2208) Bluing Reagent (760-2037)
FANCD2	1:200	Abcam	Benchmark Ultra Ventana	Rabbit	AB108928	CC2	48 min	OptiView DAB IHC Detection Kit-Ventana Medical Systens
